# Graphene-Modified Co-B-P Catalysts for Hydrogen Generation from Sodium Borohydride Hydrolysis

**DOI:** 10.3390/nano12162732

**Published:** 2022-08-09

**Authors:** Xinlei Jia, Zhen Sang, Lixian Sun, Fen Xu, Hongge Pan, Chenchen Zhang, Riguang Cheng, Yuqian Yu, Haopan Hu, Li Kang, Yiting Bu

**Affiliations:** 1 School of Material Science & Engineering, Guangxi Key Laboratory of Information Materials, Guangxi Collaborative Innovation Center of Structure and Property for New Energy and Materials, Guilin University of Electronic Technology, Guilin 541004, China; 2 School of Mechanical & Electrical Engineering, Guilin University of Electronic Technology, Guilin 541004, China; 3 School of New Energy Science and Technology, Xi’an Technological University, Xi’an 710021, China

**Keywords:** NaBH_4_, graphene oxide, catalytic activity, hydrolysis

## Abstract

Sodium borohydride (NaBH_4_) is considered a good candidate for hydrogen generation from hydrolysis because of its high hydrogen storage capacity (10.8 wt%) and environmentally friendly hydrolysis products. However, due to its sluggish hydrogen generation (HG) rate in the water, it usually needs an efficient catalyst to enhance the HG rate. In this work, graphene oxide (GO)-modified Co-B-P catalysts were obtained using a chemical in situ reduction method. The structure and composition of the as-prepared catalysts were characterized, and the catalytic performance for NaBH_4_ hydrolysis was measured as well. The results show that the as-prepared catalyst with a GO content of 75 mg (Co-B-P/75rGO) exhibited an optimal catalytic efficiency with an HG rate of 12087.8 mL min^−1^ g^−1^ at 25 °C, far better than majority of the findings that have been reported. The catalyst had a good stability with 88.9% of the initial catalytic efficiency following 10 cycles. In addition, Co-, B-, and P-modified graphene showed a synergistic effect improving the kinetics and thermodynamics of NaBH_4_ hydrolysis with a lower activation energy of 28.64 kJ mol^−1^. These results reveal that the GO-modified Co-B-P catalyst has good potential for borohydride hydrolysis applications.

## 1. Introduction

Since the first industrial revolution, the overconsumption of fossil energy has created issues of air pollution and energy storage [1]. Therefore, the development of new renewable green and efficient energy has become an urgent matter for the future development of society and the economy. Hydrogen is expected to be a fossil energy alternative, which relies on its outstanding features of the nonemission of pollutants and high efficiency [2]. In general, there are several ways, such as photocatalysis, biomass decomposition, chemical hydrides hydrolysis, to produce hydrogen [3,4]. In the method described above, hydrogen-rich compound hydrolysis, such as NaBH_4_ [5] and ammonia borane (NH_3_BH_3_) [6], has been considered as a convenient, economical, and efficient way to produce hydrogen.

NaBH_4_ is rich in hydrogen (10.8 wt%), environmentally friendly, safe, and non-flammable, which can be employed for producing hydrogen through hydrolysis reactions [7]. The hydrolysis reaction occurs through the following reactions:

NaBH_4_ + 2H_2_O → NaBO_2_ + 4H_2_ + heat (217 kJ mol^−1^) (1)

During this process, four moles of H_2_ can be produced by one mole of NaBH_4_. In particular, NaBH_4_ and water each provide 50% of the hydrogen. [8]. In addition, the byproduct NaBO_2_ can be collected to reproduce NaBH_4_, which shows a sustainable development value. However, the NaBH_4_ hydrolysis reaction exhibits sluggish kinetics in the solutions. Previous reports have proved that selecting an appropriate catalyst can significantly improve the HG rate. Noble metal-based catalysts (Pt [9,10], Ru [11,12], and Pd [13,14]) have shown positive catalytic performance. However, their scarce storage and high price limit the related practical applications. Transition metal catalysts (Co [15,16], Ni [17], and Co-Ni [18]) with inferior cost and relatively good catalytic activity have been broadly investigated for hydrogen production from NaBH_4_. In addition, transition metals combined with heteroatoms, such as boron (B) and phosphorus (P), could further enhance the catalytic activity [19,20]. For example, Patel et al. reported that the transition-metal borides (e.g., CoB, NiB) exhibited superior catalytic activities due to the mutual electronic interaction between boron and transition metals (Co or Ni), thus preventing them from oxidation and protecting the active metal center. [21]. Chen et al. prepared cobalt–phosphorus (Co-P) catalysts and investigated their catalytic efficiency in alkaline sodium borohydride solutions. The Co-P catalyst showed favorable hydrolysis performance with a low activation energy, which was attributed to the improvement of the catalytic performance by the appropriate amount of P doping [22]. So far, catalysts including Co-P [22], Ni-B [23], Co-W-B [24], Co-Ni-B [25], Co-B-P [26], etc., have been extensively researched and have shown good catalytic performance. Although these catalysts possess preferable catalytic activity, they usually show a low cycle stability. To address the above issue, selecting a suitable matrix, such as MOFs [27], porous carbon [28], MWCNTs [29], SiO_2_ [30], and γ-Al_2_O_3_ [31], which possesses a high specific surface area to support the active metals, can effectively improve the catalytic performance. Recently, graphene with excellent physical and chemical characteristics has been researched, making it an ideal carrier material to support metal clusters [32,33]. The large specific surface area can not only improve the distribution of metal clusters, thereby exposing more catalytically active sites for catalysis reaction, but it can also suppress the aggregation issue during the catalytic process, thus presenting a superior catalytic performance.

In this study, we successfully prepared the graphene modified Co-B-P catalysts through chemical in situ reduction. The structural characteristics and catalytic efficiency of the Co-B-P/xrGO (x = 25, 50, 75, 100) catalysts were studied. The Co-B-P/75rGO catalyst exhibited an optimal catalytic performance with an average HG rate of 12,087.8 mL min^−1^ g^−1^. In addition, the effects of the GO content and heteroatom types on the catalytic activity of NaBH_4_ hydrolysis were also studied. The excellent hydrogen generation performance is attributed to the fact that the large specific surface area of graphene oxide can better disperse Co-B-P clusters and thus expose more active sites. Meanwhile the elemental B and P doping exhibits a synergistic catalytic effect. This is because the presence of GO increases the specific surface area for uniform dispersion of Co-B-P clusters on the surface of the GO, thus exposing more catalytically active sites for the hydrolysis reaction.

## 2. Materials and Methods

### 2.1. Materials

High purity flake graphite (300 mesh), sulfuric acid, hydrochloric acid, hydrogen peroxide, sodium nitrate, potassium permanganate, sodium borohydride, sodium hydroxide, cobalt chloride hexahydrate, and sodium hypophosphite monohydrate were obtained from Alfa Aesar Co., Ltd. (Tianjin, China). The chemicals used were analytical reagent. All experiments used ultrapure water.

### 2.2. Synthesis of GO

We prepared the GO materials using a modification of the Hummers method [34]. First, concentrated H_2_SO_4_ (60 mL), NaNO_3_ (2 g), and flake graphite (2 g) were mixed at 5 °C to obtain a solution. Subsequently, 12 g of KMnO_4_ was slowly added to the above solution, and the solution was heated to 35 °C for 7 h with magnetic stirring. Then, 200 mL ice water and 15 mL H_2_O_2_ were added in turn to the mixed solution, until the mixed solution changed from brown to bright yellow. Next, the mixed solution was repeatedly washed with hydrochloric acid and deionized water until the pH was 7 to obtain the GO solution. Finally, the GO was obtained after freeze-drying for 72 h.

### 2.3. Catalyst Preparation

The Co-B-P/75rGO was obtained through chemical in situ reduction synthesis. First, GO (75 mg), CoCl_2_·6H_2_O (5 mmol), and NaH_2_PO_2_·H_2_O (30 mmol) were dispersed into 20 mL ultrapure water with sonication for 30 min. Next, 20 mL solution containing an appropriate amount of NaBH_4_ (30 mmol) was slowly dropped into the reaction solution with intense agitation. After being aged in an ice water bath for 10 h, the Co-B-P/75rGO catalysts were obtained after washing with water, washing with ethanol, and drying. For comparison, a Co-B-P cluster without GO was prepared under the same conditions. In addition, we controlled the addition of GO to be 25, 50, and 100 mg, and the obtained corresponding composites were labeled as Co-B-P/xrGO (x = 25, 50, and 100), respectively. The comparison samples of CoB, CoP, and CoBP without GO were prepared under the same conditions.

### 2.4. Catalyst Characterization

The Co-B-P/xrGO catalyst structures were analyzed by X-ray diffraction (XRD). The chemical structures of the catalyst were characterized by Fourier transform infrared (FTIR) spectroscopy. The elemental valence states of the Co-B-P/xrGO catalysts were determined by X-ray photoelectron spectroscopy (XPS). The morphologies of the Co-B-P/xrGO catalysts were determined by scanning electron microscopy (SEM). The degree of graphitization of the Co-B-P/xrGO catalysts was analyzed by Raman microscope (Raman spectra). The specific surface areas of the Co-B-P/xrGO catalysts were calculated by the Brunauer–Emmett–Teller (BET) method. The bulk elemental composition of Co, B, and P in the as-prepared Co-B-P/xrGO catalysts was measured via inductive coupled plasma–optical emission spectroscopy (ICP-OES).

### 2.5. Hydrogen Generation Measurement

The catalytic efficiency of Co-B-P/xrGO in alkaline NaBH_4_ solution was evaluated by a laboratory fabricated self-assembled drainage device [35]. The amount of hydrogen produced was determined by the volume of water drained, and the hydrogen generation rate was calculated through tracking the volume of water expelled at regular periods. Firstly, 0.1 g of Co-B-P/xrGO was added to a dry 125 mL wide-mouth flask. Then, 10 mL of a solution (1.5 wt% NaBH_4_ and 5 wt% NaOH) was placed into a wide-mouth flask through a 10 mL capacity syringe, and an appropriate amount of sodium hydroxide solution inhibited the NaBH_4_ self-hydrolysis reaction. The hydrogen generation efficiency of the catalyst hydrolysis was tested at different temperatures, and the reaction activation energy (Ea) was evaluated by the exponential law of reaction rate. After the hydrolysis test, the catalyst was washed with water and vacuum dried for 10 h. Then, the catalyst was tested for durability by adding 10 mL of fresh NaBH_4_ solution as described above.

## 3. Results and Discussion

### 3.1. Catalyst Characterization

**Figure 1:**
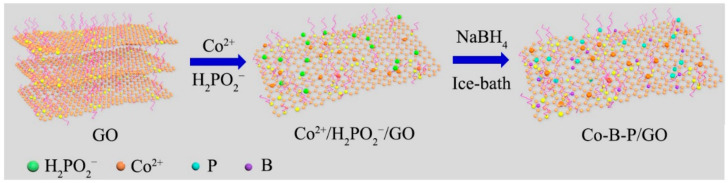
The illustration of the synthetic route of Co-B-P/xrGO.

The Co-B-P/xrGO was prepared through the chemical in situ reduction method (Figure 1) [36]. In a typical procedure, GO material was prepared by a modified Hummers method and distributed in ultrapure water under ultrasonic conditions. Subsequently, Co^2+^ was anchored on the GO surface by the electrostatic adsorption. After adding the NaH_2_PO_2_·H_2_O and NaBH_4_ solution, GO was reduced to rGO, and Co-B-P clusters formed on the rGO surface [37].

**Figure 2:**
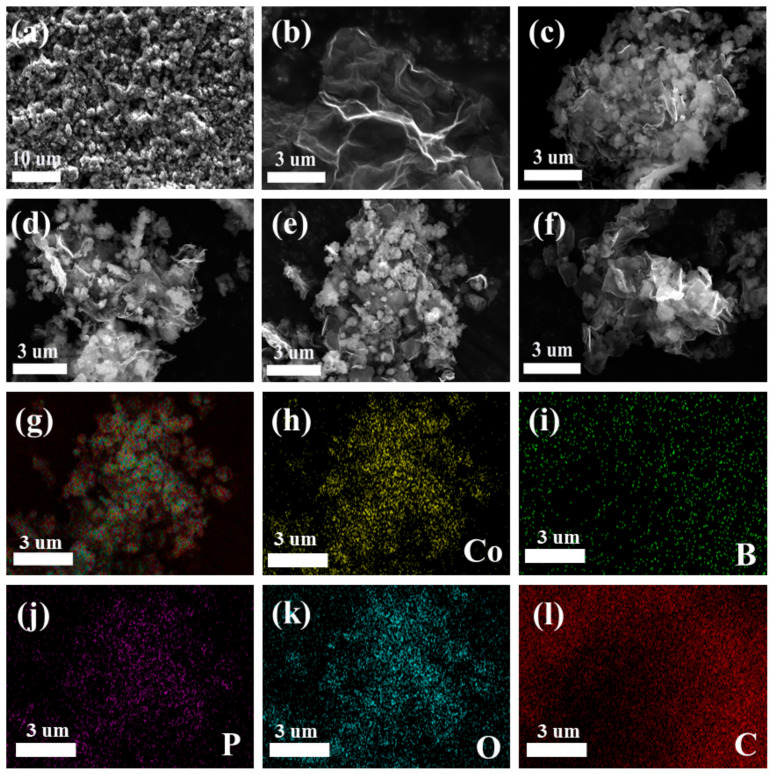
SEM images of (**a**) Co-B-P, (**b**) GO, (**c**–**f**) Co-B-P/xrGO (x = 25, 50, 75, and 100), and EDX mapping images of (**g**) Co-B-P/75rGO, (**h**) Co, (**i**) B, (**j**) P, (**k**) O, and (**l**) C in Co-B-P/xrGO.

The microscopic morphology and nanostructure of the catalysts were characterized through SEM. The prepared pure Co-B-P alloy catalyst was agglomerated in a granular morphology (Figure 2a), which was ascribed to the exothermic nature of the catalyst during the preparation process. Figure 2b shows that the GO was successfully synthesized by the modified Hummers method with a typical pleated-sheet morphology. To overcome the aggregation issue, GO with a typical pleated structure can act as a matrix material to disperse the Co-B-P clusters [38]. For exploring the effect of the GO addition on the catalytic performance, catalysts with different contents of GO were prepared. Figure 2c–f show the morphologies of Co-B-P/xrGO (x = 25, 50, 75, and 100), respectively. All the SEM images showed that the metal clusters were tightly anchored to the surface of the reduced graphene. Increasing the content of GO means the larger specific surface area can be used to provide a larger space for the dispersion of Co-B-P clusters. The Co-B-P clusters tended to grow uniformly on the surface of the reduced graphene. However, when the addition content was 100 mg, the redundant reduced graphene wrapped around leading to the aggregation issue of Co-B-P clusters. Among them, Co-B-P/75rGO exhibited an optimal morphology with Co-B-P clusters tightly and uniformly anchored on the surface of the reduced graphene. This structure can expose more active sites for the catalytic reaction, which was verified in subsequent hydrolysis catalysis measurements [29]. In addition, the EDX spectra (Figure 2g–l) showed that the Co, B, P, O, and C elements were uniformly dispersed in the Co-B-P/75rGO catalyst.

**Figure 3:**
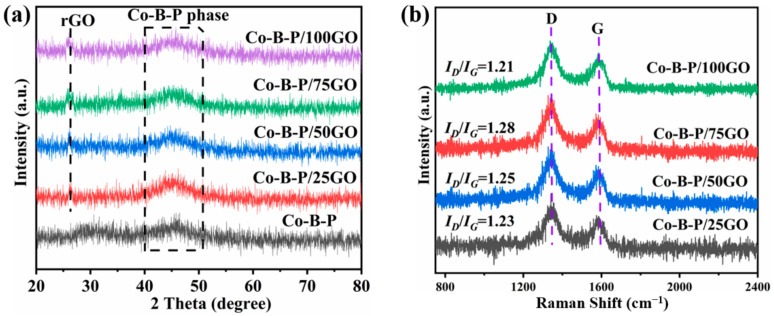
(**a**) XRD patterns of Co-B-P and Co-B-P/xrGO catalysts; (**b**) Raman spectra of Co-B-P/xrGO catalysts (x = 25, 50, 75, and 100).

The XRD patterns and Raman spectra of Co-B-P/xrGO were measured as shown in Figure 3a. A broad diffraction peak near 2θ = 45° corresponded to the Co-B and Co-P phases, indicating that the as-prepared catalysts were a typical amorphous structure [39,40], and the addition of GO would not affect the amorphous structure of the catalyst. The peaks around 26.0° belonged to the (002) plane of reduced graphene, indicating that the GO was reduced. The short-range ordered and long-range disordered amorphous structures are generally considered to have an unsaturated surface coordination, which has been proved to be beneficial for catalytic hydrolysis [21]. The characteristic peaks of the D-band and G-band were observed near 1350 and 1580 cm^−1^, as shown in Figure 3b. The ratio of the strength of the D band to the G band represents the disorder of the carbon-based hybrid material [41]. Experimental results showed that with the addition of GO, the I_D_/I_G_ values of all catalysts were greater than 1.00; in particular, Co-B-P/75rGO (I_D_/I_G_) reached 1.28. The I_D_/I_G_ value indicated that Co-B-P/75rGO had more defects, which can anchor more metal and metal-like clusters to improve the catalytic performance. The following performance test experiments also confirmed this conclusion.

**Figure 4:**
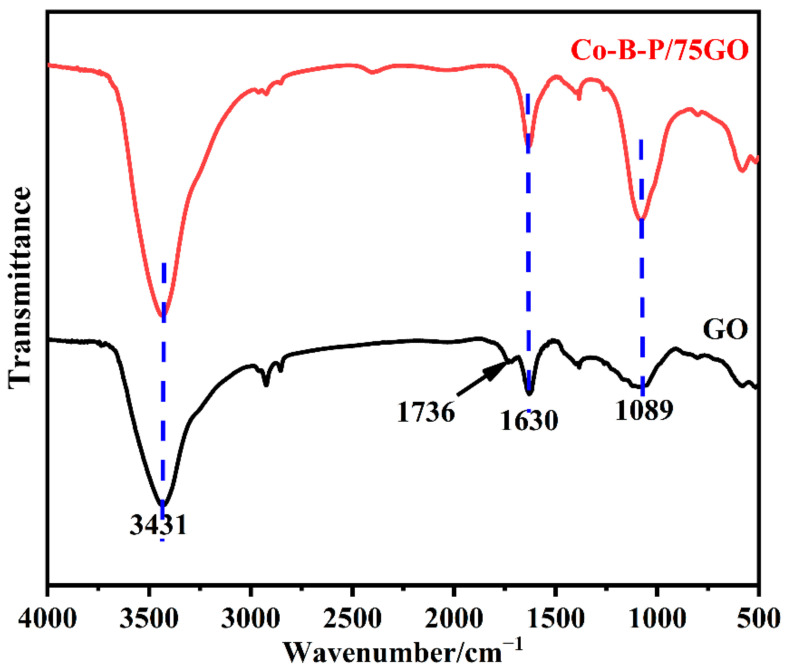
FTIR spectra of GO and CO-B-P/75rGO.

The chemical structures of GO and the Co-B-P/75rGO were characterized by FTIR (Figure 4). For the spectra of GO, the peak of the -OH stretching vibration of water molecules appeared at 3431 cm^−1^ [42]. The characteristic peaks at 1736, 1630, and 1089 cm^−1^ were observed for the -COOH stretching vibration, C=C bond skeleton vibration, and C-O-C vibration of GO, respectively [42]. The considerable numbers of oxygen-containing groups contained in the GO were produced during the oxidation of the graphite with a strong oxidizer, which can easily absorb metal ions. The FTIR spectrum of Co-B-P/75rGO was similar to the GO; yet, the peak near 1736 cm^−1^ disappeared. We ascribed this to the addition of H_2_PO_2_^−^ and BH_4_^−^, which acted as reducing agents to reduce the GO to reduced graphene (rGO) [39]. These experimental results show that the Co-B-P/75rGO catalyst was successfully synthesized.

**Figure 5:**
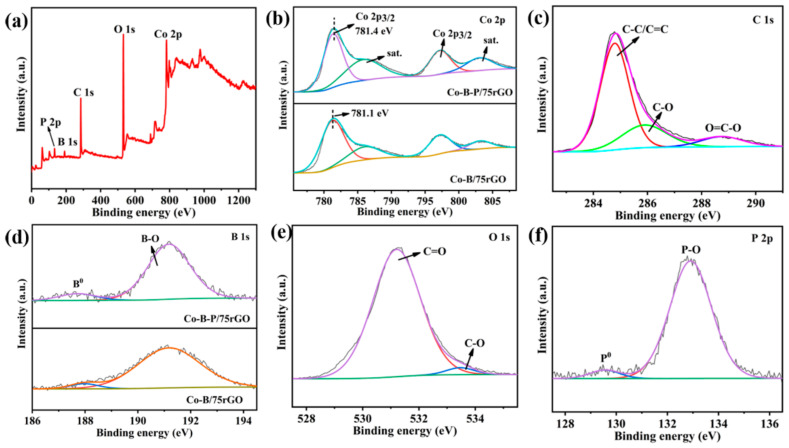
XPS analysis of Co-B-P/75rGO: (**a**) full spectrum, (**b**) Co 2p, (**c**) C 1s, (**d**) B 1s, (**e**) O 1s, and (**f**) P 2p.

The surface interactions and electronic states of the Co-B-P/75rGO catalyst were investigated by XPS. In the XPS spectrum of Co 2p (Figure 5b), the two major peaks at 781.4 and 797.2 eV were Co 2p 3/2 and Co 2p 1/2, respectively [43], while two satellite peaks were observed at 786.5 and 803.1 eV, indicating the presence of elemental Co and the oxidized state of Co in the catalyst [44]. The C 1s spectrum (Figure 5c) showed three peaks located at 284.8, 285.9, and 288.7 eV, which belonged to the C-C/C=C, C-O, and O=C-O groups of rGO, respectively [45]. The peaks of B 1s at 187.7 and 191.2 eV were attributed to boron in the elemental and oxidized states, respectively. The elemental boron was positively shifted by 1.2 eV compared to the pure boron (186.5 eV) binding energy [46]. This was due to the transfer of electrons from boron to cobalt, filling the empty d-orbitals of cobalt (Figure 5d). In the O 1s XPS spectrum, two peaks located at 531.6 and 533.0 eV were ascribed to -C=O and -C-O, respectively (Figure 5e). In addition, two distinctive characteristic peaks near 129.5 and 132.9 eV in the full spectrum of element P (Figure 5f) were attributed to the presence of P^0^ and P-O, respectively [29]. Due to the high electronegativity of P, the binding energy of P^0^ was negatively shifted by 0.7 eV compared to pure P (130.2 eV) [47]. Apparently, as shown in Figure 2, the binding energy of cobalt in Co-B-P/75rGO was positively shifted by 0.3 eV compared to that in Co-B/75rGO.These experimental results suggest that there was an interaction between Co, B, and P, which is more favorable for catalysis.

**Figure 6:**
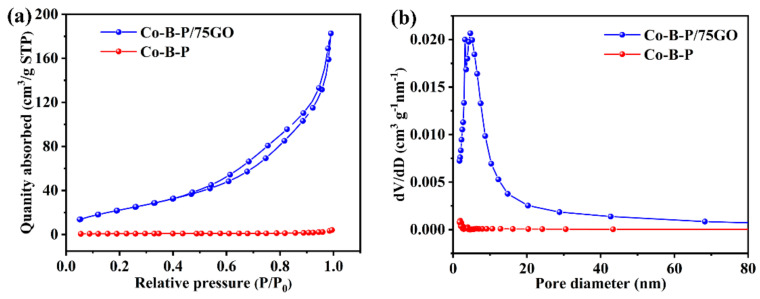
Nitrogen sorption isotherms (**a**) and pore-size distributions (**b**) for the Co-B-P and Co-B-P/75rGO catalysts.

The specific surface area and surface pore characteristics of the catalysts were tested by an Autosorb-iQ analyzer. According to the IUPAC classification, both curves in Figure 6a show hysteresis back loops, which were apparently type IV isotherms, indicating that both catalysts had a mesoporous characteristic [48]. The mesoporous channels are beneficial to the diffusion and contact between catalyst and reactant [49]. In addition, according to Table 1, the specific surface area of the Co-B-P/75rGO catalyst increased from 3 m^2^/g to 89 m^2^/g as the GO was added. Compared with pure Co-B-P, the total pore volume of Co-B-P/75rGO was increased, and the average pore diameter of 12.0 nm decreased to 9.0 nm. The addition of GO significantly increased the specific surface area for uniform distribution of Co-B-P clusters; thus, the composite catalyst offered more active sites for catalyzing the hydrolysis.

### 3.2. Effect of Different Types of Catalysts

**Figure 7:**
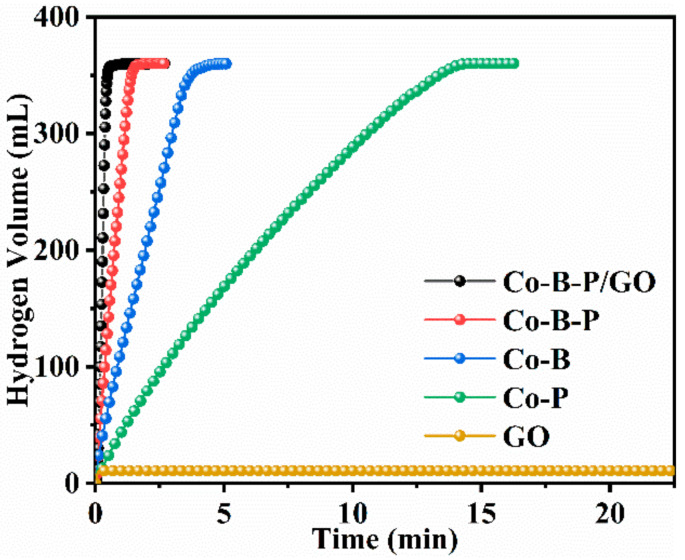
Hydrogen volume versus time for GO, Co-P, Co-B, Co-B-P, and Co-B-P/rGO (batch system, 25 °C, 1.5 wt% NaBH_4_ + 5 wt% NaOH, 0.1 g catalyst).

In order to evaluate the properties of the catalyst, performance tests with different comparison samples were carried out. Figure 7 shows the hydrogen production per unit time of sodium borohydride hydrolysis catalyzed by the GO, Co-P, Co-B, Co-B-P, and Co-B-P/75rGO catalysts, and the magnitude of the slope represents the different superior and inferior performances. The experimental results showed that pure GO had less catalytic performance when used for NaBH_4_ hydrolysis. Moreover, the combination of Co elements with heteroatoms (e.g., B and P) presented better catalytic performance than pure Co-based catalysts, which is due to the addition of heteroatoms forming electronic interactions with Co, thereby enhancing the catalytic behavior [21]. Based on the above conclusion, the Co-B-P catalyst with two heteroatoms exhibited a better performance than Co-B and Co-P catalysts because of the synergistic effect between Co, B, and P. Moreover, after combining Co-B-P with GO, the Co-B-P/rGO catalyst presented the optimal catalytic activity and has a higher competitive advantage over previously reported catalysts (Table 2). This is because the presence of GO increased the specific surface area for the uniform dispersion of the Co-B-P clusters on the surface of GO, thus exposing more catalytically active sites for the hydrolysis reaction [38]. Therefore, our further research was based on the Co-B-P/rGO catalyst.

### 3.3. Effect of GO Amount

**Figure 8:**
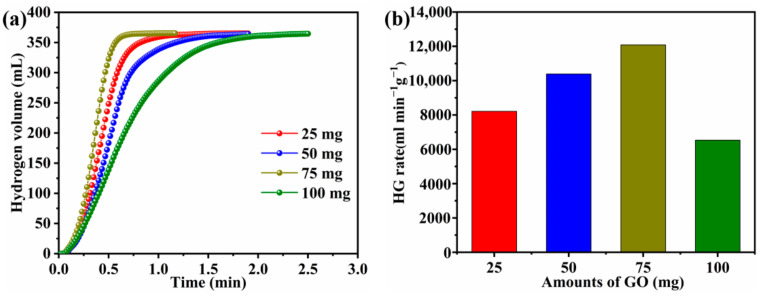
Hydrogen volume versus reaction time for the as-prepared catalysts (**a**); the histogram of the H_2_ generation rate versus the additive amount of GO (**b**) (batch system, 25 °C, 1.5 wt% NaBH_4_ + 5 wt% NaOH, 0.1 g catalyst).

The appropriate amount of carrier plays a crucial role in the synthesis of catalysts. The effects on Co-B-P/rGO catalysts with different amounts of GO (25, 50, 75, and 100 mg) for the catalytic activity of NaBH_4_ were also investigated. The hydrolysis of NaBH_4_ experiments showed that, with an increase in the amount of GO, the HG rate first increased and then decreased. The Co-B-P/75rGO sample with 75 mg GO presented an optimal catalytic performance with the HG rate of 12,087.8 mL min^−1^ g^−1^ (Figure 8a,b). Previous studies have proved that metal clusters play a major role in the hydrolysis NaBH_4_ reaction. The chemical composition of the prepared catalysts with different amounts of GO were determined by ICP–OES (Table 3). The results showed that the Co-B-P/75rGO catalyst had the highest Co content (61.79%), which also corresponded to the results of the hydrolysis experiment. Combined with the textural and surface morphology analysis, there were two factors for the superior performance of the Co-B-P/75rGO. First, the optimal content of GO supplied sufficient specific surface area for uniform distribution of Co-B-P/75rGO and provided more active sites for catalysis reaction [57]. Meanwhile, B and P heteroatoms doping led to a higher electron density in the active site of the catalyst, thus exhibiting a better catalytic performance.

### 3.4. Effect of Catalyst Amount

**Figure 9:**
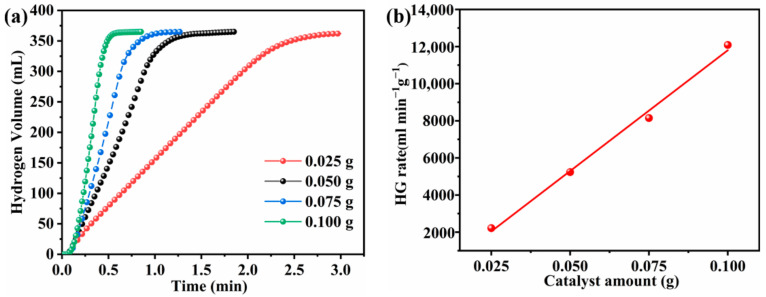
(**a**) Effect of catalyst loadings on the HG rate (batch system, 25 °C, 1.5 wt% NaBH_4_ + 5 wt% NaOH); (**b**) HG rate versus catalyst dosage.

In order to investigate the relationship between the catalyst amount and catalytic performance, four groups of different masses of Co-B-P/75rGO (25, 50, 75, and 100 mg) were tested for hydrolysis performance (Figure 9a). Each test reached the theoretical capacity of hydrogen volume, and the HG rate became increasingly faster with the increase in catalyst dosage. A linear relationship between the two can be seen in Figure 9b. This indicates that the Co-B-P/75rGO catalyst’s catalyzing hydrogen production from NaBH_4_ was characterized by first-order reaction kinetics.

### 3.5. Effect of NaBH_4_ Concentration

**Figure 10:**
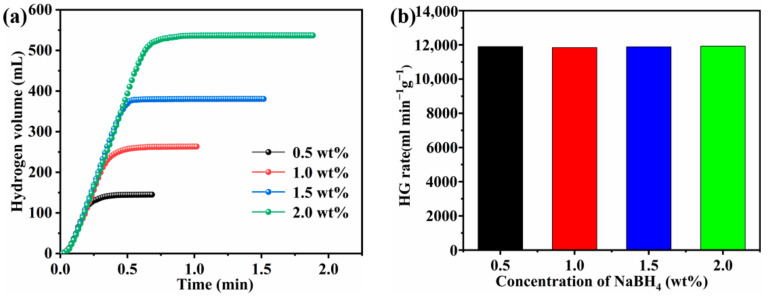
(**a**) Effect of NaBH_4_ concentrations on the HG rate (batch system, 25 °C, 5 wt% NaOH, 0.1 g of catalyst); (**b**) HG rate versus NaBH_4_ concentration.

The effect of NaBH_4_ concentration on hydrogen generation was studied under the condition of 0.1 g Co-B-P/75rGO catalyst and 25 °C (Figure 10a). The generated hydrogen volume was gradually increased to the theoretical capacity after increasing the NaBH_4_ concentration from 0.5 wt% to 2.0 wt%. In addition, Figure 10b shows that the HG rate remained nearly identical as the NaBH_4_ concentration increased. The insignificant change in HG rate indicated that the concentration of NaBH_4_ did not affect the HG reaction, showing zero-order reaction kinetics [58].

### 3.6. Kinetic Studies at Different Temperatures

**Figure 11:**
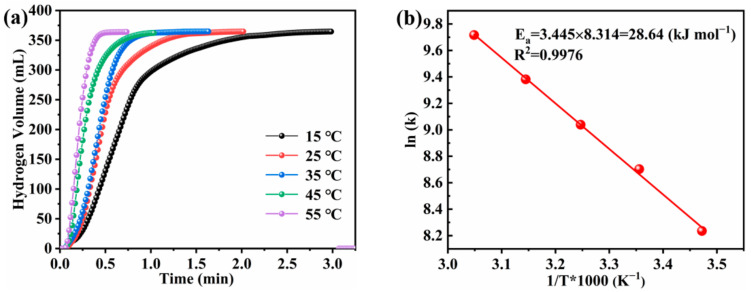
(**a**) Hydrogen generation kinetics curves and (**b**) Arrhenius plot obtained using 1.5 wt% NaBH_4_ and 1.0 wt% NaOH solution and employing Co-B-P/75rGO as a catalyst at different solution temperatures.

The HG rate of the Co-B-P/75rGO catalyst was measured under standard conditions. The temperature was controlled from 15 °C to 55 °C with 10 °C as a gradient. Figure 11a shows that high temperature had a significant promotion effect on the rate of hydrogen production. The total HG volume reached theoretical capacity at different temperatures. The formula is shown below:

(2) $${k=k}_{0}\cdot \exp\left(\frac{E_{a}}{\mathrm{RT}}\right)$$

where k_0_ is the rate constant (mL min^−1^ g^−1^), E_a_ is the activation energy (kJ mol^−1^), T is the reaction temperature (K), and R is the gas constant (8.314 kJ mol^−1^ K^−1^). Figure 11b shows the Arrhenius plot of ln k and the reciprocal of the absolute temperature (1/T). According to the slope of the fitting line, the Ea of the hydrolysis reaction in this study was calculated to be 28.64 kJ mol^−1^, which is lower than most previous reports in the literature (Table 2). The favorable catalytic activity was ascribed to the presence of GO, which promoted the uniform dispersion of Co-B-P clusters and exposed more catalytically active sites for the hydrolysis. Meanwhile the synergistic effect of the GO and Co-B-P clusters was also conducive to the hydrolysis activity of NaBH_4_.

### 3.7. Reusability Performance

**Figure 12:**
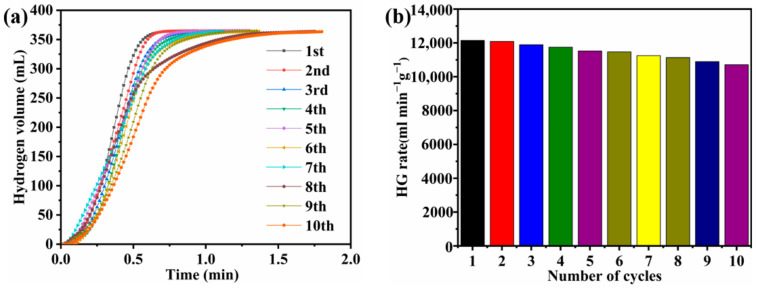
(**a**) Reusability of Co-B-P/75rGO with 0.1 g catalyst and 1.5 wt% NaBH_4_ + 5 wt% NaOH solution at 25 °C; (**b**) HG rate bar chart of catalyst used 10 times.

The cycle stability of catalysts is critical in practical applications. Therefore, NaBH_4_ was hydrolyzed 10 times with Co-B-P/75rGO catalyst in the same conditions. Figure 12 shows the variation in the catalytic hydrogen production efficiency of the Co-B-P/75rGO catalyst with the number of cycles. It can be observed that the HG rate decreased slightly as the cycle time increased. The HG rate still maintained 88.9% of the initial rate after 10 cycles, which shows better stability compared to other previously reported cobalt-based catalysts (Table 2). The decline in the catalytic activity may be due to the active clusters being reunited during each cycle. In addition, the produced boride byproducts (such as B_α_O_β_(OH)_γ_ and B_x_O_y_·nH_2_O) were adsorbed on the catalyst surface during the catalysis process, thereby decreasing the HG rate [59].

## 4. Conclusions

In summary, a series of Co-B-P/xrGO catalysts were achieved using a chemical in situ reduction method and were employed for NaBH_4_ hydrolysis. The experimental results showed that Co-B-P/xrGO had a strong effect on the catalytic behaviors, in which the Co-B-P/75rGO presented an optimal HG rate (12,087.8 mL min^−1^ g^−1^) and lower activation energy (28.64 kJ mol^−1^). The satisfied catalytic performances were due to the uniform dispersion of clusters and the synergistic catalytic effect between Co, B, and P. In addition, the repeatability test results showed that 88.9% of the initial catalytic efficiency could be maintained following 10 cycles, indicating that the catalyst had a good cycle stability. The above findings suggest that the Co-B-P/75rGO catalyst has great promise for producing hydrogen via chemical hydrate hydrolysis.

## Figures and Tables

**Table 1:** Textural parameters of the Co-B-P and Co-B-P/75rGO catalysts.

Catalyst	Specific Surface Area (m^2^ g^−1^)	Pore Volume (cm^3^ g^−1^)	Average Pore Diameter (nm)
Co-B-P	3	0.01	12.0
Co-B-P/75rGO	89	0.28	9.0

**Table 2:** The Co-B-P/75rGO catalyst was compared with those previously reported in the literature.

Sample	Maximum HG Rate (mL min^−1^ g^−1^)	E_a_ (kJ mol^–1^)	Number of Cycles	Cyclic Stability	References
Co@3DGO	4394	37.42	5	54.0%	[50]
Co@GO	5955	64.87	5	73.0%	[51]
Co-P	1647.9	47.0	5	31.0%	[39]
CoO-Co_2_P	3940	27.4	4	60.0%	[52]
Co@N MGC-500	3575	35.2	20	82.5%	[53]
Cu-Co-P/γ-Al_2_O_3_	1115	47.8	6	66.0%	[54]
Co-P/CNTs-Ni foam	2430	49.94	8	74.0%	[55]
Co-B-10CNTs	12,000	23.5	5	64.0%	[29]
Co-O-P	4850	63	5	78.0%	[56]
Co-B-50GO	14,430	26.2	5	81.5%	[40]
Co-B-P/75rGO	12,087.8	28.64	10	88.9%	This work

**Table 3:** The chemical composition of the prepared catalysts with different amounts of GO were determined by ICP–OES.

Catalyst	Amount of Co (wt%)	Amount of B (wt%)	Amount of P (wt%)
Co-B-P/25GO	35.80	0.04	16.48
Co-B-P/50GO	40.2	1.02	12.62
Co-B-P/75GO	61.79	2.51	5.50
Co-B-P/100GO	34.65	0.72	14.34

## Data Availability

Not applicable.
